# Post-diagnostic C-reactive protein and albumin predict survival in Chinese patients with non-small cell lung cancer: a prospective cohort study

**DOI:** 10.1038/s41598-019-44653-x

**Published:** 2019-05-31

**Authors:** Jin-Rong Yang, Jia-Ying Xu, Guo-Chong Chen, Na Yu, Jing Yang, Da-Xiong Zeng, Min-Jing Gu, Da-Peng Li, Yu-Song Zhang, Li-Qiang Qin

**Affiliations:** 1 Department of Nutrition and Food Hygiene, School of Public Health, Soochow University, 215123 Suzhou, China; 2 State Key Laboratory of Radiation Medicine and Protection, School of Radiation Medicine and Protection, Soochow University, 215123 Suzhou, China; 3 Department of Oncology, the Second Affiliated Hospital of Soochow University, 215004 Suzhou, China; 4 Department of Clinical Nutrition, the First Affiliated Hospital of Soochow University, 215031 Suzhou, China; 5 Department of Respiration, the First Affiliated Hospital of Soochow University, 215031 Suzhou, China; 6 Department of Oncology, the First Affiliated Hospital of Soochow University, 215031 Suzhou, China

**Keywords:** Risk factors, Respiratory tract diseases

## Abstract

Non-small cell lung cancer (NSCLC) is the most commonly diagnosed lung cancer and is associated with poor prognosis. This study aimed to analyze if serum C-reactive protein (CRP), albumin (Alb), and CRP/Alb ratio could provide prognostic information in patients with NSCLC. 387 patients with primary NSCLC were included in this analysis. Cox proportional hazards regression was used to estimate hazard ratio (HR) and 95% confidence interval (CI) of death with adjustment for some potential confounders. The multivariate regression analyses revealed the statistically significant associations of decreased survival of patients with NSCLC with elevated CRP, decreased Alb, and elevated CRP/Alb ratio. The HRs of mortality were 1.56 (95% CI: 0.80–3.04) and 2.64 (95% CI: 1.35–5.16) for patients in the second and the highest tertiles of CRP (*P*-trend = 0.003). For albumin, the HR was 0.50 (95% CI: 0.29–0.85) for the normal group. The CRP/Alb ratio strongly predicted the survival of patients in the highest tertile with a fourfold risk of dying compared with those in the lowest tertile (HR = 4.14, 95% CI: 2.15–7.98). The subgroup analysis according to various patient characteristics confirmed these associations. In conclusion, serum CRP, albumin, and CRP/Alb ratio are predictive of survival for Chinese patients with NSCLC.

## Introduction

Lung cancer is among the most common causes of cancer-related death and is associated with poor treatment outcome^1^. Non-small cell lung cancer (NSCLC) is the most diagnosed lung cancer. The majority of patients with NSCLC are usually diagnosed at later stages when curative treatments are unavailable^2^. In this regard, identifying prognostic factors is important because they can contribute to clinical decision making and help individualize treatments for this heterogeneous patient population. A number of prognostic factors in NSCLC have been described. In a systematic review of literature published between 1990 and 2001, Brundage *et al*.^3^ found 169 prognostic factors described in 887 studies on predicting the survival of patients with NSCLC. However, most of these factors are not readily available in routine practice.

Systemic inflammation has long been associated with carcinogenesis, tumor proliferation, and dissemination^4^. Inflammation may also significantly contribute to the prognostic assessment of patients with NSCLC. C-reactive protein (CRP), a routinely measured marker of chronic inflammation, is a reliable biomarker for perioperative management because it can aid in the early detection of surgical site infection^5^. Furthermore, CRP is an acute reactant protein that is increasingly expression in the presence of infection, trauma, tissue necrosis, tumor, and several types of inflammatory diseases. CRP is an important prognostic indicator in patients with several malignancies, including urological^6^, pancreatic^7^, hepatocellular^8^, and colorectal cancers^9^. In addition, some studies^10–15^ indicated that elevated serum CRP levels may predict poor survival in patients with NSCLC. However, other studies demonstrated that elevated CRP levels are unrelated to low survival^16,17^.

Another substantial aspect that is closely linked to the survival of patients with cancer is nutritional status; that is, one third of deaths are caused by malnutrition rather than the cancer itself^18^. Albumin (Alb) reflects nutritional state and response to inflammation, and is associated with the treatment outcome of NSCLC. Serum albumin is an important prognostic factor for survival of patients with NSCLC^19–21^. Some new indeices based on albumin have been reported for predicting the survival of patients with lung cancer, such indices include prognostic nutritional index^22^, Glasgow prognostic score^23^, fibrinogen and albumin score^24^, albumin and neutrophil combined prognostic grade^25^, and albumin-to-fibrinogen ratio^26^. If CRP and albumin can predict the survival of patients with NSCLC, then their combination may better predict the outcome than the use of CRP or albumin alone. CRP/Alb ratio is an independent prognostic factor in patients with pancreatic^27^, nasopharyngeal^28^, colorectal^29^ and esophageal cancers^30^. CRP/Alb ratio may also predict for disease progression and death in patients with NSCLC^31–33^. However, the evidence is still limited in China, where the incidence and mortality of lung cancer are relatively higher than those in other countries worldwide^13,17,33^. Hence, we hypothesized that elevated CRP, decreased albumin, and elevated CRP/Alb ratio are associated with poor survival of patients with NSCLC. We prospectively tested the hypothesis by using the Suzhou Lung Cancer Survival (SLCS) study.

## Results

### Characteristics of patient with NSCLC

For the 387 patients included in this study, 241 were male and 146 were female, and mean age of these patients were 62 years. A total of 105 deaths occurred during a median follow-up of 19.1 months. Baseline characteristics of the patients by tertiles of CRP level are presented in Table 1. Higher CRP level was associated with male, higher white blood cell count, lower BMI and albumin, ever smoking, later cancer stage and non-resection. Baseline characteristics according to albumin concentration are presented in Table 2. A low albumin concentration was associated with male patients, high CRP and white blood cell count, low BMI, and current smoking habits. The other characteristics of the patients by CRP level and albumin concentration are presented in Supplementary Materials Tables 1 and 2, respectively.

**Table 1:** The relationship between C-reactive protein and clinicopathological characteristics in NSCLC patients.

Variables	Total (N = 387)	C-reactive protein level (mg/L)			*P*-value
		<5.61 (N = 134)	5.61–8.58 (N = 124)	>8.58 (N = 129)	
Age (years)	387	62.10 ± 10.87	61.99 ± 11.34	63.09 ± 9.09	0.076
Sex					0.004
Male sex	241	70 (52.2)	78 (62.9)	93 (72.1)	
Female sex	146	64 (47.8)	46 (37.1)	36 (27.9)	
Smoking					0.002
Never smoker	183	76 (56.7)	63 (50.8)	44 (34.1)	
Former smoker	102	26 (19.4)	36 (29.0)	40 (31.0)	
Current smoker	102	32 (23.9)	25 (20.2)	45 (34.9)	
BMI (kg/m^2^)	387	23.67 ± 3.64	22.66 ± 3.12	21.59 ± 2.91	<0.001
Tumor type					0.431
AC	259	96 (71.7)	84 (67.7)	79 (61.2)	
SCC	96	27 (20.1)	30 (24.2)	39 (30.3)	
unknown	32	11 (8.2)	10 (8.1)	11 (8.5)	
Cancer stage					0.011
Stage I/II/III	158	54 (40.3)	65 (52.4)	39 (30.2)	
Stage IV	215	75 (56.0)	55 (44.4)	85 (65.9)	
Unknown	14	5 (3.7)	4 (3.2)	5 (3.9)	
Treatment					0.016
Resection	169	67 (50.0)	59 (47.6)	43 (33.3)	
Other therapy	178	51 (38.1)	58 (46.8)	69 (53.5)	
No therapy	40	16 (11.9)	7 (5.6)	17 (13.2)	
Albumin (g/L)					<0.001
<35	54	2 (1.5)	7 (5.6)	45 (34.9)	
≥35	333	132 (98.5)	117 (94.4)	84 (65.1)	
WBC (10^9/L)	387	5.84 ± 2.66	6.55 ± 4.06	7.88 ± 3.56	<0.001

BMI, body mass index; AC, adenocarcinoma; SCC, squamous cell carcinoma; WBC, white blood cell count.

**Table 2:** The relationship between albumin and clinicopathological characteristics in NSCLC patients.

Variables	Total (N = 387)	Albumin <35 g/L (N = 54)	Albumin ≥35 g/L (N = 333)	*P*-value
Age (years)	387	63.83 ± 10.94	62.16 ± 10.37	0.276
Sex				0.002
Male sex	241	44 (81.5)	197 (59.2)	
Female sex	146	10 (18.5)	136 (40.8)	
Smoking				0.005
Never smoker	183	15 (27.8)	168 (50.5)	
Former smoker	102	17 (31.5)	85 (25.5)	
Current smoker	102	22 (40.7)	80 (24.0)	
BMI (kg/m^2^)	387	21.70 ± 2.83	22.81 ± 3.41	0.024
Tumor type				0.852
AC	259	35 (64.8)	224 (67.3)	
SCC	96	15 (2.8)	81 (24.3)	
unknown	32	4 (7.4)	28 (8.4)	
Cancer stage				0.170
Stage I/II/III	158	16 (29.6)	142 (42.6)	
Stage IV	215	35 (64.8)	180 (54.1)	
Unknown	14	3 (5.6)	11 (3.3)	
Treatment				0.309
Resection	169	19 (35.2)	150 (45.1)	
Other therapy	178	30 (55.6)	148 (44.4)	
No therapy	40	5 (9.2)	35 (10.5)	
CRP (mg/L)				<0.001
<5.61	134	2 (3.7)	132 (39.6)	
5.61–8.58	124	7 (13.0)	117 (35.2)	
>8.58	129	45 (83.3)	84 (25.2)	
WBC (10^9/L)	387	9.32 ± 4.63	6.33 ± 3.16	<0.001

BMI, body mass index; AC, adenocarcinoma; SCC, squamous cell carcinoma; CRP, C-reactive protein; WBC, white blood cell count.

### Associations of death with CRP, albumin and CRP/Alb ratio in patients with NSCLC

**Figure 1:**
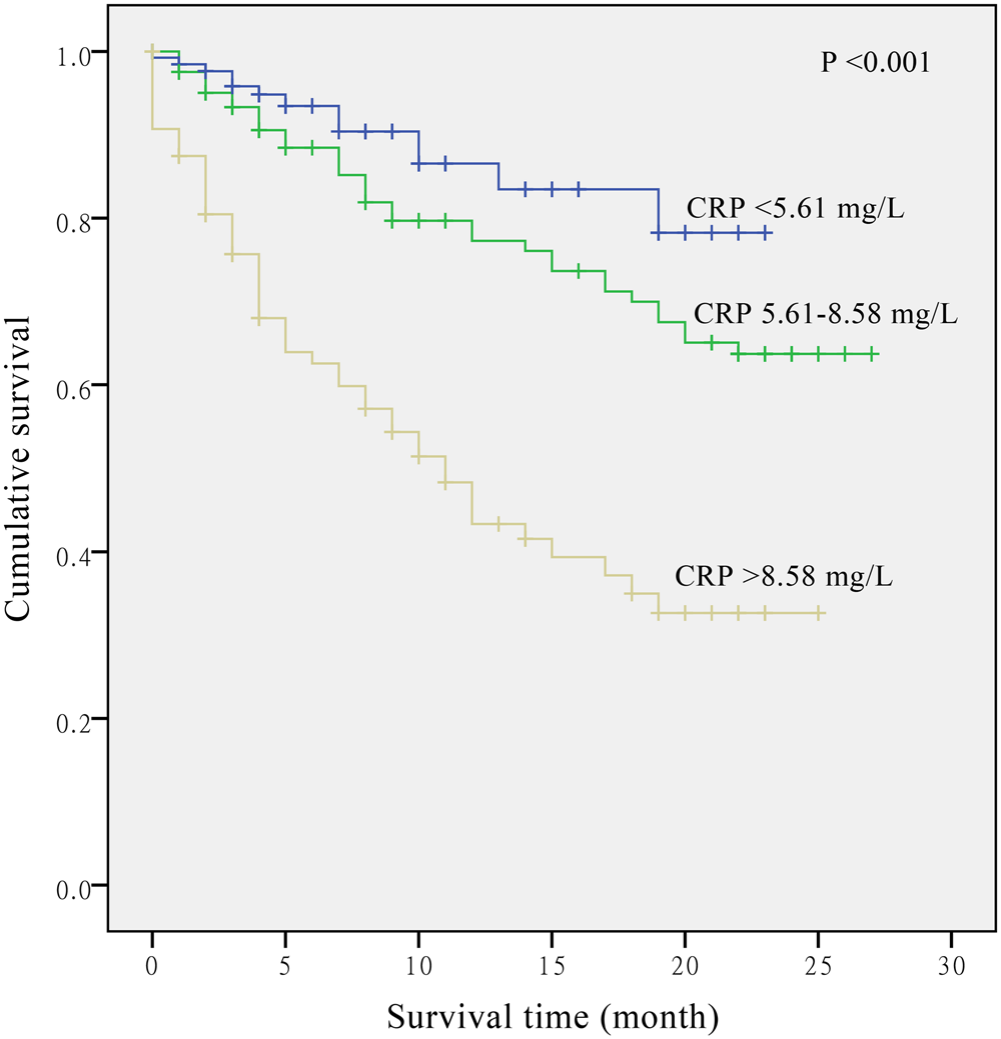
The relationship between C-reactive protein (CPR) and overall survival in patients with non-small cell lung cancer.

**Figure 2:**
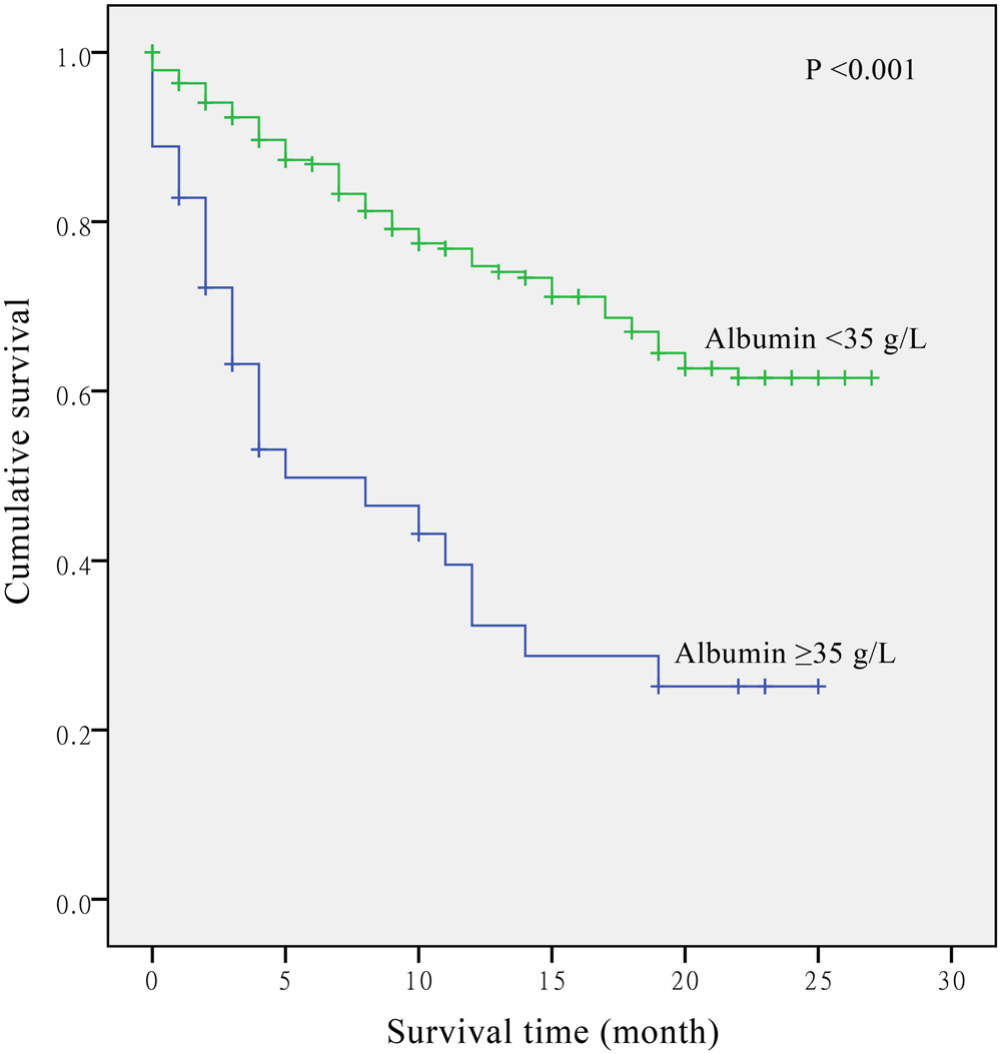
The relationship between albumin and overall survival in patients with non-small cell lung cancer.

Results of multivariate analysis with adjustments for different confounders are presented in Table 3. An increased CRP concentration was significantly associated with increased risk of death. With the basic adjustment in model 1, compared with the patients in the lowest tertile, the HRs of mortality were 1.61 (95% CI: 0.83–3.10) and 3.32 (95% CI: 1.73–6.40) for those in the second and the highest tertiles (*P*-trend < 0.001). In addition, compared with patients having low albumin level, the HR of mortality was 0.51 (95% CI: 0.31–0.82) for the group of patients with clinically normal albumin. Further adjustment of other clinical and pathological factors showed similar results for CRP and albumin (model 2). Additional adjustment for white blood cell count and patient history of chronic disease only slightly attenuated the association (model 3: top *vs*. bottom tertile of CRP HR = 2.64; 95% CI: 1.35–5.16, *P*-trend = 0.003; albumin ≥35 g/L *vs*. <35 g/L HR = 0.50; 95% CI: 0.29–0.85). Figures 1 and 2 show that the hazard of mortality for CRP (*P* < 0.001) and albumin (*P* < 0.001) were proportional, and patients with elevated CRP or decreased albumin had an increased risk of death.

**Table 3:** COX proportional hazards regression overall model of CRP, albumin and CRP/Alb ratio.

Variables	N	Model 1		Model 2		Model 3	
		HR (95% CI)	*P*-trend	HR (95% CI)	*P*-trend	HR (95% CI)	*P*-trend
**Multivariate analysis for CRP and albumin**							
CRP (mg/L)			<0.001		0.001		0.003
<5.61	134	1		1		1	
5.61–8.58	124	1.61 (0.83–3.10)		1.59 (0.82–3.09)		1.56 (0.80–3.04)	
>8.58	129	3.32 (1.73–6.40)		2.84 (1.46–5.49)		2.64 (1.35–5.16)	
Albumin (g/L)			0.006		0.002		0.011
<35	54	1		1		1	
≥35	333	0.51 (0.31–0.82)		0.45 (0.27–0.74)		0.50 (0.29–0.85)	
**Multivariate analysis for CRP/Alb ratio**							
CRP/Alb ratio			<0.001		<0.001		<0.001
<0.14	148	1		1		1	
0.14–0.22	110	2.30 (1.18–4.49)		2.16 (1.10–4.28)		2.19 (1.11–4.34)	
>0.22	129	5.16 (2.73–9.78)		4.68 (2.45–8.95)		4.14 (2.15–7.98)	

Alb, albumin; HR, hazard ratio; CI, confidence interval; CRP, C-reactive protein;

Model 1 includes age at baseline interview, sex, body mass index, family history of cancer, patient history of chronic obstructive pulmonary disease, smoking status and drinking habit.

Model 2: model 1 plus tumor type, cancer stage and treatment.

Model 3: model 2 plus history of chronic liver disease and white blood cell count.

**Figure 3:**
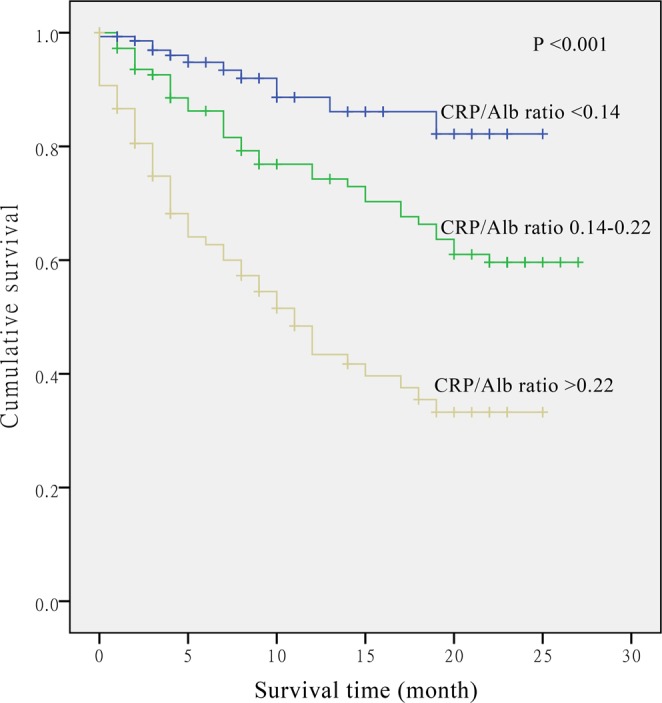
The relationship between C-reactive protein/Albumin (CRP/Alb) ratio and overall survival in patients with non-small cell lung cancer.

With the basic adjustment in model 1, the HRs of mortality were 2.30 (95% CI: 1.18–4.49) and 5.16 (95% CI: 2.73–9.78) for patients in the second and highest tertiles of the CRP/Alb ratio (*P*-trend < 0.001), compared with those in the lowest tertile. Further adjustment of other clinical and pathological factors yielded similar results (model 2). Additional adjustment for white blood cell count and patient history of chronic disease only slightly weakened the association (model 3: HR = 4.14; 95% CI: 2.15–7.98; *P*-trend < 0.003) (Table 3). Figure 3 shows the proportional hazards by CRP/Alb ratio (*P* < 0.001).

### Associations of death with CRP and albumin in subgroup analysis

The results of subgroup analysis according to various patient characteristics confirmed these associations (Table 4). The increased CRP or decreased albumin level was associated with increased risk of death. Notably, the association between CRP and death varied obviously with age and tumor type, whereas in the stratified analyses of cancer stage and other characteristics, the trend did not have much fluctuation. The association between albumin and mortality varied substantially when stratified by cancer stage and tumor resection history.

**Table 4:** COX proportional hazards regression stratified models of death and C-reactive protein, albumin.

Variables		Death/Total	C-reactive protein (mg/L) Hazard ratio (95% CI)				Albumin (g/L) Hazard ratio (95% CI)		
			<5.61	5.61–8.58	>8.58	*P-*trend	<35	≥35	*P*-value
Age (years)	<60	29/138	1	4.48 (0.78–25.86)	6.09 (1.17–31.73)	0.056	1	0.22 (0.07–0.65)	0.006
	≥60	76/249	1	1.39 (0.66–2.95)	2.62 (1.21–5.66)	0.006	1	0.49 (0.26–0.94)	0.031
Sex	Male	79/241	1	1.58 (0.64–3.88)	2.78 (1.15–6.71)	0.01	1	0.49 (0.27–0.88)	0.017
	Female	26/146	1	1.96 (0.65–5.94)	2.73 (0.86–8.68)	0.118	1	0.83 (0.16–4.21)	0.821
Smoking	Never	35/183	1	2.13 (0.76–5.93)	2.80 (0.93–8.41)	0.118	1	0.76 (0.23–2.56)	0.656
	Ever	70/204	1	1.37 (0.54–3.48)	2.67 (1.09–6.57)	0.009	1	0.45 (0.24–0.83)	0.011
Tumor type	AC	58/259	1	1.45 (0.62–3.38)	1.98 (0.85–4.64)	0.133	1	0.37 (0.18–0.78)	0.009
	SCC	35/96	1	2.64 (0.51–13.50)	5.77 (1.05–31.67)	0.032	1	0.28 (0.09–0.86)	0.027
Cancer stage	I/II/III	26/158	1	0.97 (0.26–3.63)	3.99 (0.99–16.08)	0.011	1	1.87 (0.36–9.62)	0.453
	IV	76/215	1	2.11 (0.91–4.90)	3.06 (1.34–7.01)	0.015	1	0.53 (0.28–0.98)	0.042
Resection	Yes	28/169	1	1.11 (0.34–3.64)	3.90 (1.22–12.47)	0.005	1	2.74 (0.60–12.53)	0.193
	No	77/218	1	1.66 (0.72–3.83)	2.20 (0.95–5.1)	0.094	1	0.24 (0.13–0.45)	<0.001

CI, confidence interval; AC, adenocarcinoma; SCC, squamous cell carcinoma.

Model includes age at baseline interview, sex, body mass index, family history of cancer, patient history of chronic obstructive pulmonary disease, smoking status and drinking habit, tumor type, cancer stage, treatment, history of chronic liver disease and white blood cell count.

Note: Exclude stratification factor when the model was running.

## Discussion

To the best of our knowledge, this study was first to simultaneously investigate CRP, albumin, and CRP/Alb ratio in relation to the survival of Chinese patient with NSCLC. The results demonstrate that both elevated CRP and decreased albumin levels were significantly associated with poor survival among Chinese patients with NSCLC. Moreover, an increased CRP/Alb ratio was more strongly predictive of mortality. Our findings might suggest that the combination of systemic inflammation and nutritional status affects outcome in patients with NSCLC.

Chronic inflammation, which is an emerging focus of cancer research in recent years, may play a key role in carcinogenesis and tumor progression^34^. Persistent inflammatory state produces various inflammatory cytokines in response to tissue necrosis and the presence of tumor cells, which has an important effect on cancer progression^35^. CRP, a typical inflammation marker, is an acute-phase protein produced in the liver. Its main biologic function is to recognize pathogens and damaged cells in the host and to mediate their elimination by recruiting component system and phagocytic cells^36^. Our findings support the positive associations between CRP and risk of mortality in NSCLC patients^37,38^. CRP level was classified into two groups in a number of previous studies^10–15^, and only French study^39^ observed a dose-response association between CRP and mortality with a 1.8-fold higher risk of death among stage I and II but not stage III and IV NSCLC patients when comparing the highest with the lowest group of CRP (>20 mg/L vs. undetectable). In present study of Chinese NSCLC patients, a dose-dependent relationship was also found between CRP and death regardless of cancer stage.

Upon stratification according to tumor type, a strong positive association was observed between the CRP and the mortality of patients with squamous cell carcinoma, whereas the association in patients with adenocarcinoma was relatively weak. Similarly, Siemes *et al*.^40^ reported that the increased risk of death was associated with higher CRP for squamous cell carcinoma of lung rather than adenocarcinoma. However, Mssago *et al*.^11^ reported that CRP was a poor independent prognostic factor for patients with non-squamous NSCLC. Thus, additional studies are needed to verify whether the relationship between CRP levels and survival among lung cancer patients vary with the histologic subtypes.

Upon examination of the association in terms of cancer stage, we classified the cancer stage into two groups (stage I/II/III and IV) due to the small number of patients with stage I/II NSCLC. CRP was positively associated with mortality in NSCLC patients regardless of cancer stage. Alifano *et al*.^39^ showed a CRP level above 20 mg/L was significantly associated with poor survival compared with undetectable CRP in patients with stage I/II unlike in patients with stage III/IV. However, other studies have reported that patients with elevated CRP levels had a poor survival in advanced NSCLC^11,13,15^. In addition, we found that CRP was positively associated with mortality in patients with tumor resection, which support the positive association between CRP and mortality in patients with resectable NSCLC^10,12,14^.

Malnutrition commonly occurs in cancer patients, especially in patients with advanced NSCLC who have an important energy expenditure caused by increased tumor metabolism^19^. Albumin concentration is inversely associated with the magnitude of systemic inflammatory response due to increased catabolism and the downregulation of hepatic synthesis by cytokines tumor necrosis factor alpha^41^. Using the clinical reference value^25^ as cutoff, a significant 0.50-fold lower risk of death was observed in patients with normal albumin level. Notably, substantial difference was found in the association between albumin and mortality when stratifying our analyses in terms of cancer stage and tumor resection status. The inverse association was limited to stage IV patients and those without cancer resection. Conversely, Li *et al*.^26^ showed that albumin could be a prognostic biomarker regardless of cancer stage, and Miura *et al*.^20^ found that preoperative albumin could predict overall survival in 556 resected NSCLC. The potential cause of these differences remains unclear, but the number of patients with early cancer stage or surgical resection may have been too small to detect a significant association in the aforementioned studies in other patient populations.

As expected, we observed a strong positive association between CRP/Alb ratio and risk of mortality in patients with NSCLC. Fairclough *et al*.^42^ proposed the concept of CRP/Alb ratio in 2009. Recent studies in other patient population also revealed that the CRP/Alb ratio was predictive of disease progression and mortality in patients with NSCLC^31–33^. A study reported the prognostic effect of CRP/Alb ratio on the survival of patients with advanced NSCLC in Korea^31^. The other two studies found that CRP/Alb ratio could predict the long-term outcome in Chinese^33^ and Japanese^32^ patients with operable NSCLC. Our study did not have specific restriction on cancer stage but also observed a strong positive association between CRP/Alb ratio and the mortality of patients with NSCLC.

The strengths of our study include its overall large sample size, comprehensive collection of various patient characteristics to allow careful adjustment for potential confounders. However, a small number of patients were involved in several subgroup analyses, which limited the statistical power. Lung cancer has long relied on testing for the molecular biomarkers, such as epidermal growth factor receptor (EGFR) and anaplastic lymphoma kinase (ALK)^43^. In the present study, we did not collect the samples to determine the driver gene mutation. However, a meta-analysis demonstrated that there were significantly increased odds of presenting the EGFR and ALK-EML4 mutations in adenocarcinomas compared to NSCLC^44^. Our ongoing patient enrollment could provide an additional opportunity to specifically investigate some of these associations in greater depth.

In conclusion, our study shows that increased CRP, decreased Alb level, and increased CRP/Alb ratio were associated with poor survival among Chinese patients with NSCLC.

## Materials and Methods

### Participant identification

The SLCS study, which was initiated in 2015, is an ongoing patient cohort for exploring long-term predictors of lung cancer survival among patients living in Southeastern China (Suzhou). This study mainly emphasizes on the potential influence of nutritional status and environmental pollutants on lung cancer survival. Patients who were diagnosed with primary lung cancer and who were 18 years old or older were included in the study. Although our inclusion criteria did not have other specific restrictions, such as status and type of medical treatments, data were carefully collected during the enrollment period and their effects on any examined association were explored throughout the analysis. The patient enrollment was started in January 2016, and 455 patients diagnosed with primary lung cancer have been included as of April 2018. Considering the small number of small cell lung cancer cases (n = 49), our analysis only included patients with NSCLC (n = 406). This study was approved by the Research Ethics Committee of Soochow University (Approval No. ESCU-2015-0002), and written informed consent was obtained from each patient. We also confirmed that all methods were performed in accordance with the relevant guidelines and regulations.

### Data and blood sample collection

In-person interviews were conducted using a structured questionnaire during recruitment to collect patients’ characteristics on demographics and habitual lifestyle, including smoking, alcohol drinking, and physical activity. Based on the collected data, smoking status was grouped into three categories (never, former, or current smoker), and drinking status was classified into two groups (yes or no). Former smokers were defined as patients who had smoked but already quitted for at least one year, and current smokers were determined as those who are still smoking at the time of investigation or those who gave up smoking for less than one year. Patients who are drinking alcohol on average of at least once a week within the six previous months were considered as patients with drinking habit (yes). Field measurements were implemented to obtain patient anthropometrics, including height, weight, and waist and hip circumstance. In addition to the patient interviews, their medical records were also reviewed to collect their clinical information, including the pathological type, cancer stage, and lung cancer treatments.

Blood biochemistry and blood cell analysis were also obtained from their test report during enrollment, including liver function, renal function and other blood indicators. Furthermore, 5 mL whole blood was drawn from the antecubital veins of each patient and then transferred into a tube with ethylenediaminetetraacetic acid during recruitment after an overnight fasting. The plasma was obtained through centrifugation (3,000 rpm at 25 °C for 10 min) within 2 h after sample collection. All samples were numbered and stored at −80 °C until further analysis.

### Measurements of biomarkers

Serum CRP, albumin, and white blood cell count were all extracted from the patient test report from two hospitals. CRP, albumin and white blood cell count were all conducted in laboratories of both institutions as routine examinations for each patient. Among the three indicators, the serum CRP level was measured through nephelometry by using a fully automatic Siemens ADVIA 2400 Biochemical Analyzer System (Siemens Healthcare Diagnosis Inc, NY, USA). The lower detection limit was 1 mg/L. The serum albumin concentration was measured through bromocresol green staining method by using an Olympus 5400 Automatic Biochemical Analyzer (Olympus Corporation, Tokyo, Japan). Furthermore, a combination of electrical impedance, radiofrequency and cytochemistry was used to determine the blood count by using the Sysmex/XE-2100 blood cell analysis line (Sysmex Corporation, Kobe, Japan).

### Patient follow-up

This study primarily determined the overall survival, which was calculated from the date of baseline questionnaire to the date of death. Follow-up was initiated from the date of patient enrollment until May 8, 2018 or until patient death from any cause. The long-term survival of patients was assessed every 6 months. Data were collected from at least one of the following sources: (1) inpatient or outpatient records from the hospital; (2) local death registration system; and (3) patient or family telephonic contact. A small proportion of patients (4.7%, n = 19) were lost during the follow-up and were excluded from the present analysis. A total of 387 patients with NSCLC were included in the present analysis.

### Statistical analysis

Continuous variables were expressed as the means with the standard deviation, and were compared using one-way ANOVA. The Chi-Square or Mann-Whitney U test was used to compare the categorical variables, which was presented as the number and percentage of patients. Albumin was grouped according to the clinical standard threshold^25^. The CRP concentration and CRP/Alb ratio were classified into tertiles.

Survival analysis was performed using the Kaplan-Meier method, and the differences were assessed using the Log-Rank test. Hazard ratios (HR) and 95% confidence intervals (CI) of NSCLC death were estimated using the Cox proportional hazards regression model. Three models with different covariates were constructed to account for potential confounders. The first model was adjusted for demographic characteristics including age at baseline interview (years), sex, body mass index (kg/m^2^), family history of cancer (yes or no), patient history of chronic obstructive pulmonary disease (yes or no), smoking status (never, former, or current smoker), and drinking habit (yes or no). The second model included the variables in the model 1 and was additionally adjusted for clinical information including tumor type (adenocarcinoma, squamous cell carcinoma, or other tumor types), cancer stage (I/II or III/IV), and treatment (with tumor resection, without resection but with other therapies such as chemotherapy and radiotherapy, without either resection, or other therapies). Considering the impact of white blood cell count on the CRP^45^ and the influence of chronic liver disease on the albumin^46^, analyses were further adjusted for the history of chronic liver disease (yes or no) and white blood cell count (continuous) in the third model.

For the analyses of CRP and CRP/Alb ratio, *P* values for the linear trends were calculated by fitting the median values of CRP and CRP/Alb ratio as continuous variables. We also constructed stratified analyses by age (<60 years vs. ≥60 years), sex, smoking status (never smoker vs. ever smoker), tumor type (adenocarcinoma vs. squamous cell carcinoma), cancer stage (I to III vs. IV), and tumor resection (yes vs. no) to assess whether the examined associations varied substantially according to these characteristics. All statistical analyses were performed using the SPSS 19.0 software. All tests were two-sided, and statistically significance was considered at *P* < 0.05.

## Supplementary information

Title page, and Suppl Table S1 and S2
