# Supplementary material for: Post-diagnostic C-reactive protein and albumin predict survival in Chinese patients with non-small cell lung cancer: a prospective cohort study
Source: Sci Rep. 2019 May 31;9:8143. doi: 10.1038/s41598-019-44653-x (PMC6544765; doi:10.1038/s41598-019-44653-x)
Supplement: Supplementary file 1 — Title page, and Suppl Table S1 and S2 [file 41598_2019_44653_MOESM1_ESM.pdf]

Post-diagnostic C-reactive protein and albumin predict survival in Chinese patients with non-small cell lung cancer: a prospective cohort study

Jin-Rong Yang [1], Jia-Ying Xu [2], Guo-Chong Chen [1], Na Yu [3], Jing Yang [4], Da-Xiong Zeng [5], Min-Jing Gu [6], Da-Peng Li [6,*], Yu-Song Zhang [3,*] & Li-Qiang Qin [1,*]

[1] Department of Nutrition and Food Hygiene, School of Public Health, Soochow University, Suzhou, 215123, China

[2] State Key Laboratory of Radiation Medicine and Protection, School of Radiation Medicine and Protection, Soochow University, Suzhou, 215123, China

[3] Department of Oncology, the Second Affiliated Hospital of Soochow University, Suzhou, 215004, China

[4] Department of Clinical Nutrition, the First Affiliated Hospital of Soochow University, Suzhou, 215031, China

[5] Department of Respiration, the First Affiliated Hospital of Soochow University, Suzhou, 215031, China.

[6] Department of Oncology, the First Affiliated Hospital of Soochow University, Suzhou, 215031, China.

Jin-Rong Yang and Jia-Ying Xu contributed equally to this work.

*Corresponding authors

Correspondence and requests for materials should be addressed to L.-Q.Q (e-mail: qinliqiang@suda.edu.cn), Y.-S.Z.(e-mail: zhangyusong19@163.com) and D.-P.L.(e-mail: lidapeng@suda.edu.cn).

Supplementary Materials Table S1.

The relationship between C-reactive protein and clinicopathological characteristics in NSCLC patients.

Variables	Total (N=387)	C-reactive protein level (mg/L)			*P*-value
		<5. 61 (N=134)	5.61-8.58 (N=124)	>8.58 (N=129)	
Anthropometrics					
Height (cm)	387	163.70±7.81	165.10±6.26	165.35±6.70	0.118
Weight (kg)	387	63.71±12.00	61.76±7.32	59.18±9.92	0.002
Waist circumstance (cm)	387	85.48±11.68	83.83±7.47	81.29±8.72	0.024
Hip circumstance (cm)	387	94.84±8.90	89.97±5.83	89.02±7.15	<0.001
Drinking habit					0.099
Yes	84	25 (18.7)	24 (19.4)	35 (27.1)	
No	303	109 (81.3)	100 (80.6)	94 (72.9)	
History of chronic liver disease					0.286
Yes	19	8 (6.0)	7 (5.6)	4 (3.1)	
No	368	126 (94.0)	117 (94.4)	125 (96.9)	
Liver function					
Direct bilirubin (μmol/L)	387	3.63±1.65	4.55±5.84	5.16±4.02	0.012
Indirect bilirubin (μmol/L)	387	7.57±3.42	6.07±2.84	6.69±4.80	0.006
Globulin (g/L)	387	28.00±3.96	28.16±4.57	30.19±7.16	0.001
Aspartate transaminase (U/L)	387	26.72±21.22	26.58±32.17	21.29±17.41	0.126
Alanine aminotransferase (U/L)	387	25.35±12.86	26.35±33.56	22.11±11.48	0.260
Kidney function					
Blood urea nitrogen (mmol/L)	387	5.34±2.77	5.23±2.45	4.97±1.93	0.456
Serum creatinine (μmol/L)	387	65.13±17.81	66.30±17.24	65.56±21.20	0.881
Uric acid (μmol/L)	387	304.29±90.77	316.65±90.53	270.99±102.05	<0.001

Supplementary Materials Table S2.

The relationship between albumin and clinicopathological characteristics in NSCLC patients.

Variables	Total (N=387)	Alb <35 g/L (N=54)	Alb ≥35 g/L (N=333)	*P*-value
Anthropometrics				
Height (cm)	387	168.82±5.52	164.36±7.16	0.017
Weight (kg)	387	60.46±9.07	61.76±10.89	0.408
Waist circumstance (cm)	387	81.36±7.32	83.79±9.68	0.196
Hip circumstance (cm)	387	89.45±5.72	91.38±7.89	0.214
Drinking habit				0.798
Yes	84	11 (20.4)	73 (21.9)	
No	303	43 (79.6)	260 (78.1)	
History of chronic liver disease				0.658
Yes	19	2 (3.7)	17 (5.1)	
No	368	52 (96.3)	316 (94.9)	
Liver function				
Direct bilirubin (μmol/L)	387	5.48±5.13	4.26±4.00	0.047
Indirect bilirubin (μmol/L)	387	6.34±4.91	6.87±3.62	0.343
Globulin (g/L)	387	30.53±8.66	28.49±4.72	0.011
Aspartate transaminase (U/L)	387	24.04±23.62	25.01±24.49	0.785
Alanine aminotransferase (U/L)	387	23.40±13.99	24.79±22.53	0.660
Kidney function				
Blood urea nitrogen (mmol/L)	387	4.91±1.79	5.23±2.50	0.368
Serum creatinine (μmol/L)	387	64.23±17.91	65.88±18.90	0.549
Uric acid (μmol/L)	387	241.48±109.66	306.29±90.91	<0.001

Alb, Albumin
